# An Interphase Microfluidic Culture System for the Study of Ex Vivo Intestinal Tissue

**DOI:** 10.3390/mi11020150

**Published:** 2020-01-30

**Authors:** Martha Baydoun, Anthony Treizeibré, Jérôme Follet, Sadia Benamrouz Vanneste, Colette Creusy, Lucie Dercourt, Baptiste Delaire, Anthony Mouray, Eric Viscogliosi, Gabriela Certad, Vincent Senez

**Affiliations:** 1 Univ. Lille, CNRS, ISEN-YNCREA, UMR 8520-IEMN, F-59000 Lille, France; 2 ISA-YNCREA Hauts de France, F-59000 Lille, France; 3 Univ. Lille, CNRS, Inserm, CHU Lille, Institut Pasteur de Lille, U1019-UMR 9107-CIIL-Centre d’Infection et d’Immunité de Lille, F-59019 Lille, France; 4 Laboratoire Ecologie et Biodiversité, Unité de Recherche Smart and Sustainable Cities, Faculté de Gestion Economie et Sciences, Institut Catholique de Lille, F-59800 Lille, France; 5 Service d’Anatomie et de Cytologie Pathologiques, Groupement des Hôpitaux de l’Université Catholique de Lille, 59000 Lille, France; 6 CNRS, Univ. Tokyo, UMI 2820 — LIMMS, F-59000 Lille, France; 7 Plateforme d’Expérimentations et de Hautes Technologies Animales, Institut Pasteur de Lille Lille, 59019 Lille, France; 8 Délégation à la Recherche Clinique et à l’Innovation, Groupement des Hôpitaux de l’Institut Catholique de Lille (GHICL), Faculté de Médecine et Maïeutique, Université Catholique de Lille, 59800 Lille, France

**Keywords:** organotypic tissue model, intestinal tract model, ex vivo microfluidic tissue culture, long term viability maintenance

## Abstract

Ex vivo explant culture models offer unique properties to study complex mechanisms underlying tissue growth, renewal, and disease. A major weakness is the short viability depending on the biopsy origin and preparation protocol. We describe an interphase microfluidic culture system to cultivate full thickness murine colon explants which keeps morphological structures of the tissue up to 192 h. The system was composed of a central well on top of a porous membrane supported by a microchannel structure. The microfluidic perfusion allowed bathing the serosal side while preventing immersion of the villi. After eight days, up to 33% of the samples displayed no histological abnormalities. Numerical simulation of the transport of oxygen and glucose provided technical solutions to improve the functionality of the microdevice.

## 1. Introduction

Organotypic tissue models, either explants or organoids, have a long history [1,2,3,4]. They complement the set of two-dimensional (2D) and three-dimensional (3D) in vitro culture techniques with the goal of offering, ex vivo, more advanced tissue functions than any current in vitro models without the difficulties inherent to in vivo studies (i.e., low throughput and high cost). Explanted tissues are likely to recapitulate the whole complexity of in vivo. They can be cultured in a controlled environment and many of them can be harvested from a single animal reducing the use of animals testing in agreement with the 3 Rs (replacement, reduction and refinement) principle. In addition, several experiments can be performed with the same donor, increasing the robustness and reproducibility of the model. However, the maintenance of the viability is challenging due to limited diffusion of molecules when the size of the solid biopsy is greater than a few hundred micrometers. The extraction and interpretation of information also remains difficult as compared with simpler models of the organ [5]. Therefore, increasing their longevity is of great interest to the biomedical community.

Organotypic models of the intestine have been used to study the role of the microbiome in health and disease [6,7], to elucidate the origin of several pathologies [8] and to screen new therapeutic molecules [9]. Important advances have been made recently in their development and validation, although none have replaced human tests [10]. They include organoid culture [11], organ-on-a-chip [12], precision-cut slice [13], and tissue explant [14]. The introduction of microfluidics and microfabrication technologies in organotypic culture has the potential to provide more relevant culture conditions through accurate control of spatial and temporal distribution of fluids, transported molecules, and physical stimuli applied [15]. These miniaturization technologies have already largely diffused the field of three-dimensional (3D) engineered culture assays [16,17,18,19]. Indeed, microflow systems have been tested with solid biopsies of different organs [5,20,21,22,23,24,25,26,27] including the intestine [28,29,30,31]. Midwoud et al. studied the perfusion of precision-cut intestinal slices in a multilayer polydimethylsiloxane (PDMS) device and retained their in vivo metabolic rate up to 8 h [28]. Costa et al. developed a 3D printed device to study porcine gut and obtained viability up to 24 h [29]. Yissachar et al. adapted the air–liquid interface culture model to a microfluidic format preventing loss of architecture of the mucosa up to 40 h [30]. Dawson et al. reported the microfluidic culture of punched human intestinal fragment where both the luminal and serosal sides were perfused with a culture medium up to 72 h [31]. The design of the culture microsystem was inspired by the method of Browning and Trier consisting of culturing the explant at the air–liquid interface (ALI) [32]. ALI culture systems offer several advantages as compared with submerged culture systems since they keep the stratified architecture of the epithelium and provide a better oxygenation and nutrients delivery to the bottom part of the explant thanks to the porosity and permeability of the supporting membrane. However, to properly feed the tissue and ensure its long-term viability, the culture media needs to be changed periodically or continuously perfused. The technological challenge with dynamic perfusion is to select a membrane material with physical properties (i.e., porosity and permeability) that, on the one hand, allows a sufficient transport of nutrients and gases to the biopsy and, on the other hand, prevents its submersion. Our group has previously succeeded in maintaining a colonic static explant, mechanically supported by a nitrocellulose (NC) porous membrane, viable for 35 days [14]. In order to make the change of the culture medium easier, we designed a microfluidic system incorporating an ALI for the biopsy culture. Due to technological difficulties, it was not possible to use NC membrane and we chose to integrate a commercial polycarbonate (PC) membrane. To understand the effects of the materials’ properties of both NC and PC materials, we implemented a numerical model taking into account the mass transport and culture medium flow. The experimental result showed that our PC membrane was less efficient than our previous NC membrane to deliver enough oxygen and nutrients to the biopsies and this was confirmed by the numerical analysis. 

## 2. Materials and Methods 

**Figure 1:**
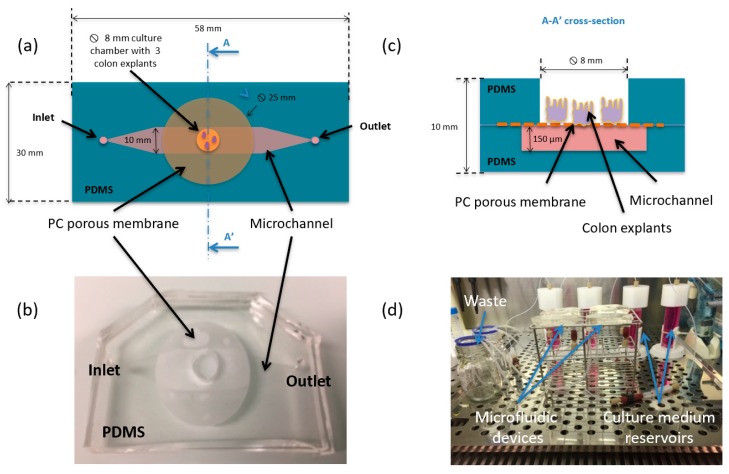
(**a**) Schematic top view of the microfluidic system made of two polydimethylsiloxane (PDMS) layers and one polycarbonate (PC) membrane; (**b**) current assembled device; (**c**) schematic drawing of the A-A’ cross-section showing the location and dimensions of the culture chamber, PC membrane, and microchannel, three colon explants are placed in each chamber; (**d**) entire set-up introduced in an incubator at 37 °C and 5% CO_2_ including 4 devices, 9 colon explants, 4 independent culture medium reservoirs and wastes.

The microfluidic device was composed of two polydimethylsiloxane (PDMS) layers separated by a PC microporous membrane (25 mm diameter and pores size 0.4 μm) (Figure 1a). The bottom layer was a replica obtained from a micromachined silicon mold. It formed a microchannel (150 μm thick and 1 cm wide) for the perfusion of the explant. The culture chamber was defined in the top layer (5 mm thick) using a punch biopsy needle (8 mm diameter) to generate a through hole. The sealing was performed by plasma activation, mechanical and thermal compression. Silicon tubing with an internal diameter of 500 μm was secured to the inlet and outlet holes by PDMS polymerization (Figure 1b). The microsystem was connected to a microfluidic flow control system (MFCS-350mb, Fluigent, Le Kremlin-Bicêtre, France) in order to bath the serosal side of the explant, while avoiding immersion of the mucosa layer (Figure 1c). Four microfluidic devices were run in parallel (one triplicate and one empty control) at 37 °C and 5% CO_2_ inside a humidified incubator at a volumetric flow rate of 10 μL/min for up to 8 days (Figure 1d). The colon explants were prepared and analyzed as previously described [14]. Three explants (9 in total) were placed in each incubation chamber. Animal protocols were approved on 3 May 2011 by the French regional ethical committee (approval number CEEA 112011). Detailed description of the device fabrication and operation is available in the Appendix A.

To understand how the membrane properties modified the tissue viability, we implemented a computational model to calculate the transport by convection and diffusion of glucose and oxygen in the bioreactor and their consumption by the cells in the tissue explant. In order to quantify the efficiency of the microsystem to maintain the viability of the tissue, we estimated the reaction efficiency for glucose and oxygen, *R_effg_* and *R_effo_*, respectively, as the average rates of glucose and oxygen consumption in the tissue divided by the maximal rate of glucose and oxygen consumption (for glucose and oxygen concentrations equal to the values set at the inlet of the microchannel).

Mass transport model: Transport of oxygen and glucose is assumed to be governed by the steady-state mass transport Equations (1) and (2) either for aqueous media or explant tissue, respectively.

(1) $$0=-u\nabla c_{i}+\beta D_{i}^{a}\nabla^{2}c_{i}$$

where *c_i_* denotes the concentration (mol/m^3^) of the species *i*, ∇ the nabla operator, $D_{i}^{a}$ the diffusion coefficient (m^2^/s) of the species *i* in the aqueous phase, *u* is the velocity of the aqueous media (m/s), and β is an empirical factor taking into account the retardation effect observed in the diffusion of molecules within the membrane (β = 1 in the culture medium and is defined in Table 1 for the PC and NC membranes).

(2) $$0=-R_{i}^{t}+D_{i}^{t}\nabla^{2}c_{i}$$

where *c_i_* denotes the concentration (mol/m^3^) of the species *i*, ∇ the nabla operator, $D_{i}^{t}$ the diffusion coefficient (m^2^/s) of the species *i* in the explant tissue, and $R_{i}^{t}$ is the reaction rate (mol/m^3^/s) of species *i* in the explant tissue. 

The reaction term is expressed with a Michaelis–Menten consumption kinetics:

(3) $$R_{i}^{t}=R_{i}^{t,\;max}\frac{c_{i}}{c_{i}+c_{i}^{max/2\;}}\delta (c_{i}>c_{th})$$

where $R_{i}^{t,\;max}$ is the maximum consumption rate of species *i* in the explant tissue, $c_{i}^{max/2}$ the Michaelis–Menten constant corresponding to the concentration of species i where consumption drops to 50% of its maximum, *c_th_* is the threshold concentration of species *i* below which the reaction term is cancelled, and δ() a step-down function to cease the consumption when *c_i_* is below *c_th_*.

Fluid dynamics model: The culture medium is an aqueous media, being considered as incompressible and flowing from the microchannel to the explant tissue through the porous membrane. We do not take into account the effect of the inlet velocity and liquid evaporation on the localization of the air–liquid interface. The Reynolds number (R_e_) is small (1 < R_e_ < 10), and thus the conservation of linear momentum can be modeled by the Stoke equation:

(4) $$\eta \nabla^{2}u=\nabla p-F$$

where *F* (kg/m^2^/s^2^) is a body force acting on the aqueous media, *η* is the dynamic viscosity (Pa·s), *u* is the aqueous media velocity (m/s), p is the pressure (kg/m/s^2^).

Dimensionless numbers: The Peclet number is defined as follows:

(5) $$P_{e}=\;\frac{Lu}{\beta D_{i}^{a}}$$

where *L* (m) is the characteristic length of the microsystem (here the membrane thickness), *u* is the average velocity of the culture medium through the membrane, *β* is the empirical coefficient for diffusion retardation effect, and $D_{i}^{a}$ is the diffusion coefficient of species *i* in the culture medium.

*R_effi_* number (*i* standing either for oxygen or glucose) is defined as the actual rate of reactant consumption (averaged over a given volume/surface of the biological tissue) and divided by the reaction rate that is measured if the reactant concentration in the tissue is uniform and equal to the value set at the micro-channel inlet:

(6) Reffi= (∑1nci)/n(∑1nci)/n+cimax/2 ci,0ci,0+cimax/2 

where *i* stands for either oxygen or glucose, n (n > 4000) is the number of nodes in the bottom part of the explant domain (i.e., half of the thickness), and c_i,0_ is the maximal concentration of either oxygen or glucose entering at the inlet of the microfluidic channel (see Table 1). C_o,0_ = 0.174 mol/m^3^ and C_g,0_ = 25 mol/m^3^.

The analytical equation giving the permeability of fibrous materials as defined by Tomadakis is as follows:

(7) $$\frac{k}{r^{2}}=\frac{\varepsilon }{8*\ln^{2}\left(\varepsilon \right)}*\frac{{\left(\varepsilon -\varepsilon_{p}\right)}^{\left(\alpha +2\right)}}{{\left(1-\varepsilon_{p}\right)}^{\alpha }*{\left[\left(\alpha +1\right)*\varepsilon -\varepsilon_{p}\right]}^{2}}$$

where *k* is the permeability (m^2^), *r* is the radius of the fiber (m), *ε* is the porosity of the porous media, *α* is a constant (0.785), and *ε_p_* is the percolation threshold (0.11).

Geometry and boundary conditions: The calculation of the distribution of glucose and oxygen have been implemented in a finite element solver (COMSOL Multiphysics, COMSOL, Genoble, France) by solving mass transport and fluid flow in two-dimensional (2D) for the different device geometries in steady-state regime. The microchannel height is T_p_ = 150 μm, the tissue explant thickness is T_e_ = 300 μm, the culture media height is T_c_ = 250 μm in the culture chamber, the diameter of the porous membrane is L_m_ = 8000 μm, the diameter of the explant tissue is L_e_ = 3600 μm, the length of the device is L_d_ = 36,000 μm. The finite element method (FEM) model is made of about 1 487 841 degrees of freedom for the PC geometry and 1 275 851 degrees of freedom for the NC geometry using the predefined ”extra fine” mesh refinement. In the mass transport model, the following boundary conditions were implemented: zero normal mass flow at side walls, continuity between culture medium, porous membrane and tissue explant, and fixed concentration for culture medium in contact with exterior. In the fluid flow model, no slip was imposed to all surface corresponding to a solid–liquid interface, a fixed velocity (between 0.01 and 10 m/s) was used for the inlet and a fixed pressure (P = 0 Pa) for the outlet. It takes about 600 s and 9.7 Gb of memory to solve the equations on an Intel Core i7-7500U CPU cadenced at 2.7 GHz with 16 Go RAM.

## 3. Results 

**Figure 2:**
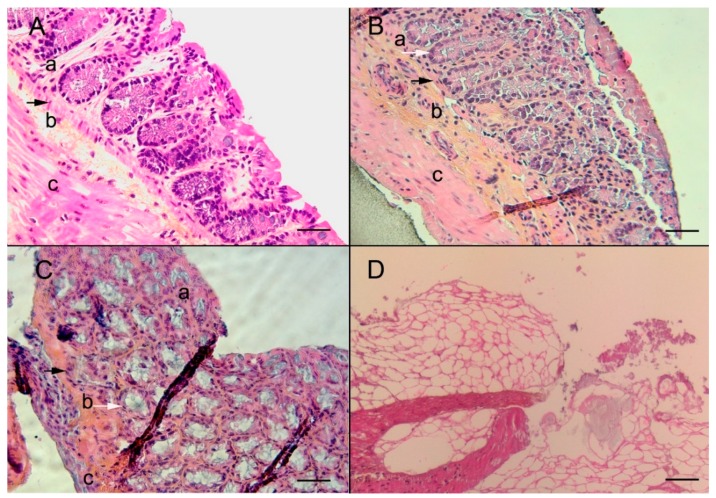
Histological analysis of the murine colonic explant in the microfluidic culture system. (**A**) Normal non-cultivated murine colon section with a characteristic prismatic epithelium (**a**), intact muscularis mucosa (black arrow), submucosa (**b**), and muscular layer (**c**) are observed, hematoxylin/eosin and safranin (HES), scale bar 40 µm; (**B**) normal colonic explant section after 8 days of microfluidic culture, the overall structure is maintained showing characteristic prismatic epithelium (**a**), muscularis mucosa (black arrow), submucosa (**b**), and muscular layer (**c**), glands are of normal size, and the basal lamina is preserved (white arrow), HES, scale bar 20 µm; (**C**) colonic explant section after 8 days of microfluidic culture, layers of the colonic section, mucosa showing some signs of necrosis (**a**), muscularis mucosa (black arrow), submucosa (**b**), and muscular layer (**c**), the basal lamina is still preserved (white arrow), HES, scale bar 20 µm; and (**D**) colonic explant section after 8 days of microfluidic culture showing a complete necrosis, HES, scale bar 20 µm.

To test the efficiency of the microfluidic culture chamber to maintain the interphase conditions, we studied the evolution of the flow rate as a function of the pressure difference between the inlet and outlet. The volume flow rate was found to increase linearly in the microchannel up to 100 mbars corresponding to a hydrodynamic resistance of 1.2 × 10^13^ kg/m^4^/s. The working pressure drop was fixed at 20 mbars to robustly prevent flowing of the culture medium through the PC membrane. The histological analysis (Figure 2), showed the following: (i) three explants presented a preserved histological organization, (ii) four explants presented signs of stress, and (iii) two explants were necrosed.

**Figure 3:**
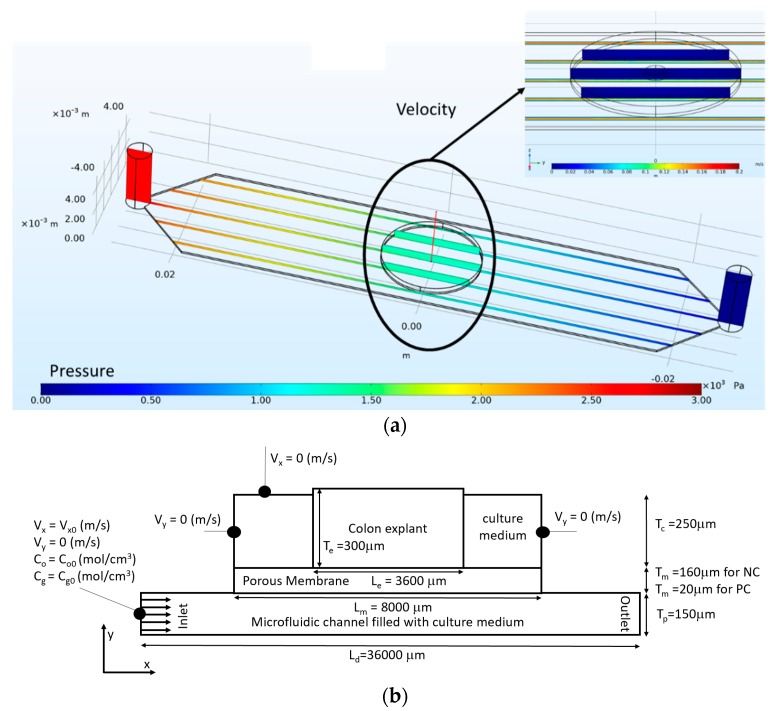
Schematic geometry of the microfluidic system. (**a**) 3D view of the internal geometry of the culture microsystem with the pressure field along the device and in insert with the velocity field in the central part; (**b**) 2D longitudinal cross-section of the geometry with details of the boundary conditions.

We studied the influence of culture medium velocity and permeability of the membrane on *R_effg_* and *R_effo_*. The channel height was fixed to 150 μm and the glucose concentration to 25 mol/m^3^ according to the composition of the DMEM-F12 medium. Oxygen concentration was fixed by the operating conditions in the incubator (0.174 mol/m^3^). In order to reduce the computing time, the phenomena responsible for mass transport and consumption were modeled in 2D. However, we simulated the laminar flow in a 3D model for a pressure drop of 20 mbars (Figure 3a) to apply the right flow boundary conditions at the inlet of the 2D model. The result shows that the average velocity of culture medium is 0.15 m/s in the widest section of the microfluidic channel (10 mm wide). Consequently, we studied the behavior of the device for inlet velocity between 0.010 and 10 m/s. The dimensions of the microsystem and boundary conditions are detailed in the 2D longitudinal cross-section in Figure 3b. The spatial distributions of glucose and oxygen were predicted by a convection-diffusion-reaction equation assuming Michaelis–Menten kinetics. The flow of the culture medium was calculated with the Stoke equation.

**Figure 4:**
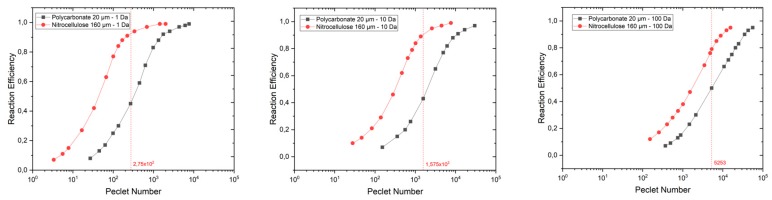
Variations of the reaction efficiency for oxygen (*R_effo_*) as a function of the Peclet number for three membrane permeabilities. (**left**) 1 Darcy; (**center**) 10 Darcy; and (**right**) 100 Darcy for a 20 μm thick PC (black curve) and 160 μm thick nitrocellulose (NC) (red curve) membranes. The vertical red line corresponds to the simulation of the experimental conditions.

In order to synthesize the results of our study, we plotted (Figure 4) the variation of *R_effo_* as a function of the Peclet (*Pe*) dimensionless number, for different membrane permeability values (between 1 and 100 Darcy). To define the range of permeability, we used the analytical model of Tomadakis giving four orders of magnitude between PC and NC membranes permeability for a given radius of the fibers [39]. Knowing the range of culture medium velocities at the microdevice inlet, we limited our analysis to three permeabilities: 1, 10, and 100 Darcy (1 Darcy = 9.869233 × 10^−13^ m^2^, a medium with a permeability of 1 Darcy permits a fluid flow of 10^−6^ m³/s with a viscosity of 10^−3^ Pa·s under a pressure gradient of 10,132,500 Pascal/m acting across an area of 10^−4^ m²). The delivery of oxygen and glucose is assumed to be achieved only through the culture medium. Thus, it depends on the inlet flow velocity and the transport properties of the membranes. The porosity values were provided by the manufacturers for both PC and NC membranes while permeability values were unknown. The combined effect of the inlet velocity and membrane permeability is represented in the Peclet number as defined above.

## 4. Discussion

We have succeeded in performing intestinal tissue explant culture in a microfluidic system keeping tissue morphology up to 192 h for 33% of the samples. Previous studies combining intestine explant cultures and microfluidics reported a maximal viability of 72 h. Many factors can influence solid biopsy viability [40]. Among them, organism size [41], donor age [42], localization of the fragment in the organ [42,43], composition of the culture medium [44], format of the culture [45] (either static or dynamic, fully immersed or mechanically supported explant at the air-culture medium interface), and duration of the transfer [46] can play key roles. Therefore, a significant variability in duration of their viability was highlighted, from several hours in Ussing chamber [47] up to 91 days [48]. 

A porous membrane provides high viability for 3D explant culture [49]. Polytetrafluoroethylene (PTFE) NC- and PC-based membranes are classical materials found in commercial inserts. The incorporation of membrane functionalities in microfluidic devices is a relatively new research area [50]. Various techniques such as gluing [51], sandwiching [52], clamping [53], or direct in situ fabrication [54] have been reported, even if they remain technologically challenging. The major difficulties are (i) the sealing step due to incompatibility between sticking properties of the various polymeric materials and thickness of the membrane (typically few microns) and (ii) the reproducibility in the fabrication to ensure constant flow through the different experiments. 

As a continuation of previous work on explant culture by our team [14], first, we tried to integrate a NC-based membrane (160 μm thick) in the microfluidic device. Although this technological approach has been reported [55,56], the achievement of a perfect sealing remains very difficult to obtain [57] and we did not succeed in preventing the immersion of the explant. Therefore, we introduced a PC membrane (20 μm thick) according to the process reported by Chueh et al. and it was very effective [58]. However, NC and PC materials do not have the same transport properties.

**Figure 5:**
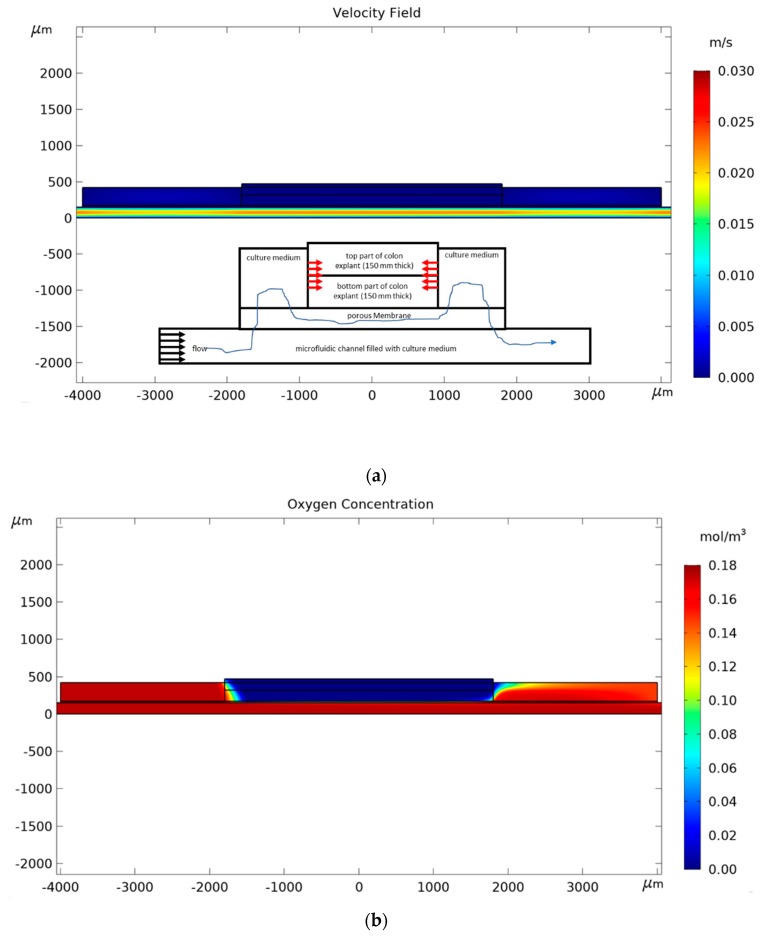

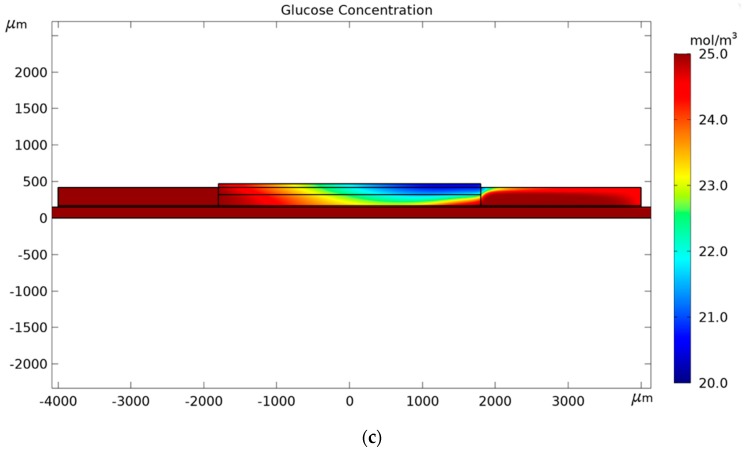
2D distribution in the microfluidic devices containing a PC membrane for a 1 Da permeability of (**a**) the culture medium velocity; (**b**) oxygen; and (**c**) glucose with an inlet flow velocity of 0.015 m/s. The insert on the velocity figure (**a**) schematizes the main flow of the culture medium around the explant and its effect on oxygen and glucose transport (red arrows accounts only for diffusion process).

**Figure 6:**
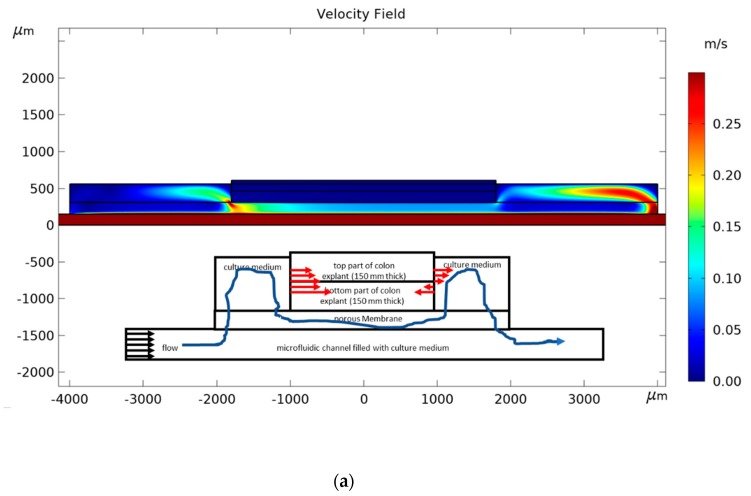

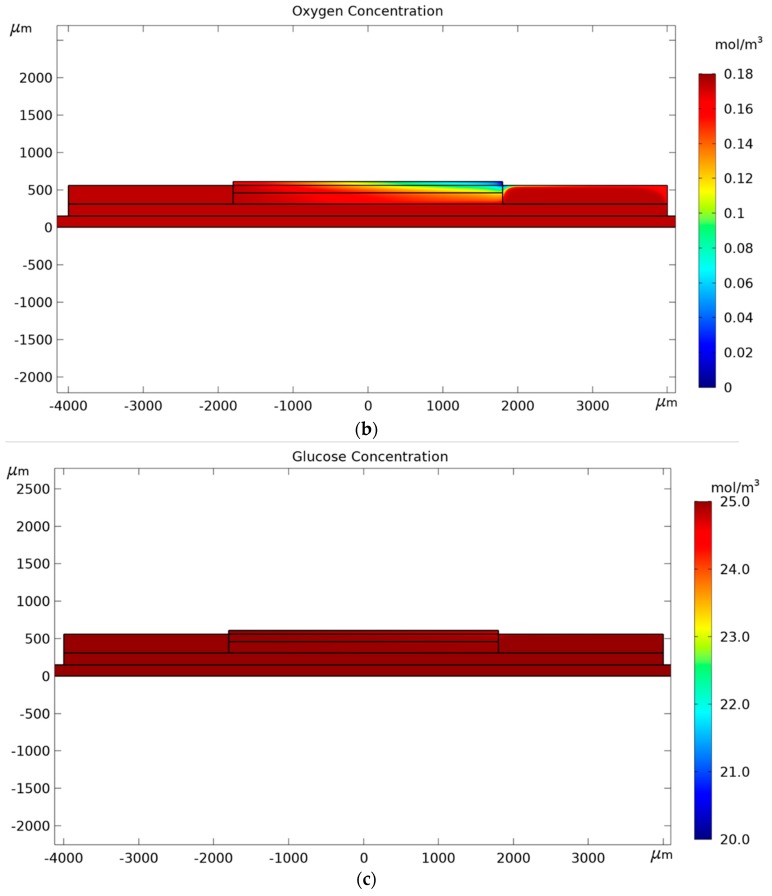
2D distribution in the microfluidic devices containing a NC membrane for a 100 Da permeability of (**a**) the culture medium velocity; (**b**) oxygen; and (**c**) glucose with an inlet flow velocity of 2 m/s. The insert on the velocity figure (**a**) schematizes the main flow of the culture medium around the explant and its effect on oxygen and glucose transport (red arrows account for both diffusion and convection process).

**Figure A1:**
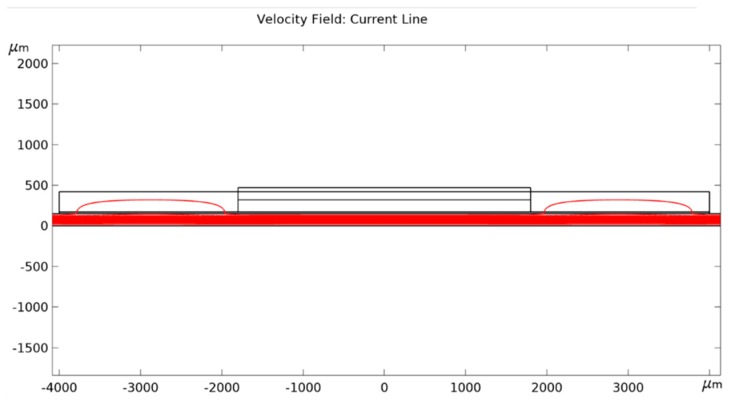
2D distribution in the microfluidic devices containing a PC membrane with a 1 Da permeability for an inlet flow velocity of 0.015 m/s.

**Figure A2:**
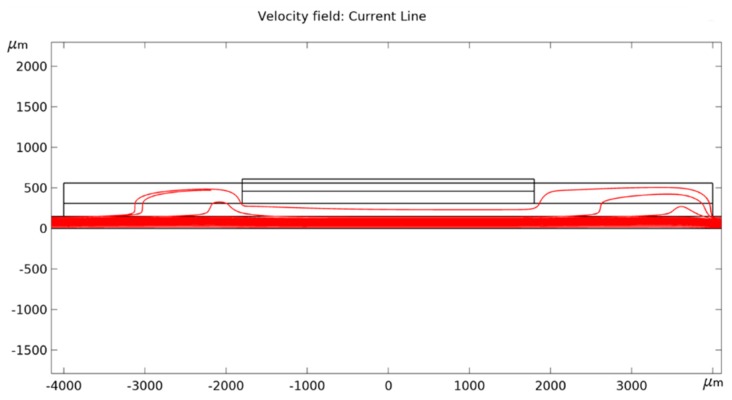
2D distribution in the microfluidic devices containing a NC membrane with a 100 Da permeability for an inlet flow velocity of 2 m/s.

Indeed, one can observe in Figure 4 that the highest permeability has the highest Peclet number for a given inlet velocity. For all permeabilities, the reaction efficiency is higher for NC than that of PC membranes. The difference in reaction efficiency for oxygen between NC and PC membranes decreases when permeability increases. Indeed, at high permeability, the transport of molecules through the membrane is performed mainly by convection reducing the effect of the difference between the diffusive properties of NC and PC membranes on the reaction efficiency. Figure 5; Figure 6 give the 2D distributions of culture medium velocity, as well as oxygen and glucose concentrations for the two extreme cases we studied, namely a device with a PC membrane having a permeability of 1 Da and an inlet flow velocity of 0.015 m/s and a device with a NC membrane having a permeability of 100 Da and an inlet velocity of 2 m/s. Additionally, the corresponding velocity line distributions for these two cases are given in Figure A1 and Figure A2. In the first case, the flow magnitude around the explant is very low that prevents convective transport of oxygen and glucose in the explant. In this configuration, the delivery of oxygen and glucose at the heart of the explant depends only on diffusion. The difference between oxygen and glucose distribution is due to the difference of gradients (0.174 mol/m^3^ for oxygen and 25 mol/m^3^ for glucose). In the case of a more permeable membrane (100 Da) and higher inlet flow rate (2 m/s), the delivery of oxygen and glucose to the explant is performed by both convection and diffusion. As a result, the amount of oxygen and glucose in the tissue is higher and the difference between the distribution of both species is lowered. For the value of the inlet flow velocity (i.e., 0.15 m/s represented by the dash vertical red line on each graph in Figure 4) used in the experiments, the reaction efficiency for the PC membrane is always lower than 0.5, while for the NC membrane it is always above 0.8. Even if the same trend is found for glucose delivery, one can see that the concentration of glucose remains quite high in the explant with a reaction efficiency always higher than 0.94 (results not shown). According to Figure 4, we conclude that it would be necessary to multiply the flow rate by almost 10 in future experiments to improve the oxygen delivery to the explants and allow a longer preservation of their morphology.

## 5. Conclusions

The majority of microfluidics models of human intestinal culture rely either on the culturing of an intestinal epithelial cell monolayer or on an organoid culture. Regardless of the importance of this type of culture, it remains limited when it comes to the in vivo microenvironment. We described an organotypic microfluidic mice colon culture model that kept the morphology of the intestinal tissues up to 192 h for 33% of the explant. Numerical simulation showed that the PC membrane is less adapted to oxygen delivery than the NC membrane and, consequently, it would be necessary, in the future, to multiply the flow rate by almost 10 to increase the rate of success with PC membrane.

## Figures and Tables

**Table 1:** Physical properties for mass transport or consumption of oxygen and glucose in the culture medium, porous membrane, and tissue explant.

Parameter	Unit	Aqueous Media	Porous Membrane	Tissue Explant	Reference
Temperature	°K	310	310	310	–
Density	kg/m^3^	993	N/A^c^	N/A^c^	–
Viscosity	Pa·s	0.7 × 10^-3^	N/A^c^	N/A^c^	–
Porosity	%	N/A^c^	79 ^a^ or 15 ^b^	N/A^c^	–
Permeability	Darcy	N/A^c^	1 to 100	N/A^c^	–
Oxygen diffusivity	m^2^/s	2.6 × 10^-9^	1.1× 10^-9 a^ or 9.4 × 10^-11 b^	2.0 × 10^-9^	[33]
Glucose diffusivity	m^2^/s	0.7 × 10^-9^	0,3 × 10^-9 a^ or 2.5 × 10^-11 b^	0.3 × 10^-9^	[34]
Retardation effect (β)	–	1	0,407 ^a^ or 0.036 ^b^	1	[35]
Max oxygen reaction rate	mol/m^3^/s	N/A^c^	N/A^c^	−0.034	[36]
Max glucose reaction rate	mol/m^3^/s	N/A^c^	N/A^c^	−0.028	[37]
Critical oxygen conc.	mol/m^3^	N/A^c^	N/A^c^	1 × 10^-4^	[36]
Critical glucose conc.	mol/m^3^	N/A^c^	N/A^c^	0.1	[37]
Initial oxygen conc.	mol/m^3^	0.174	0.174	0.174	[38]
Initial glucose conc.	mol/m^3^	25	25	25	–
M.M.^d^ constant (oxygen)	mol/m^3^	N/A^c^	N/A^c^	1 × 10^-3^	[36]
M.M.^d^ constant (glucose)	mol/m^3^	N/A^c^	N/A^c^	1 × 10^-2^	[37]

^a^ value for NC membrane, ^b^ value for PC membrane, ^c^ not applicable, ^d^ Michaelis–Menten.

## Appendix A

Microfluidic device fabrication: The master mold is made of silicon (Siltronix Silicon Prime wafers CZ (diameter 76.2 ± 0.3 mm, thickness 380 ± 25 μm, orientation <100>, type doping P-Boron, resistivity 1 to 10 Ω cm). Selective silicon etching is obtained by protection of the silicon surface with a resist mask. AZ15nXT negative photoresist (MicroChemicals, Ulm, Germany) is spin-coated to obtain a 10 μm thick layer (speed = 1500 rpm, acceleration = 3000 rpm·s^−1^, time 40 s). The substrate is soft baked for 3 min at 110 °C on a hotplate and AZ15nXT is exposed to UV radiation at λ = 365 nm for 45 s @ 10 mW. After this exposure step, a post-exposure is realized on a hotplate for 1 min at 120 °C. Exposed resist is removed by the MIF 326 developer (MicroChemicals, Germany) for 4 min and rinsed with water for 15 s. The substrate is etched with STS DRIE plasma equipment (SPTS, Newport, UK) by a deep reactive ion etching (DRIE) process (Bosch process), with C_4_F_8_ passivation and SF_6_ etching steps (C_4_F_8_ flow rate = 100 sccm, passivation time = 2.2 s, RIE/ICP power = 20 W/1500 W, SF_6_ flow rate = 450 sccm, etching time = 3 s, RIE/ICP power = 50 W/2200 W). The substrate chiller is cooled down to −10 °C in order to improve the thermal evacuation. The etching rate is 5.5 μm·min^−1^ and the etching depth is 150 μm for the bottom PDMS replica. A thin layer of ”Teflon-like” coating is deposited on the surface of the silicon substrate using a C_4_F_8_ plasma to facilitate the peeling of PDMS. The liquid mixture containing the precursor and the curing agent (10:1 (v/v)) is poured on the silicon mold and polymerized in a furnace at 70 °C for 2 h. The resulting micro-structured elastomer is peeled off by hand. The top PDMS layer is fabricated using the same protocol but on a flat silicon substrate and punched to make an 8 mm diameter through hole. The sealing of the two PDMS layers and the PC membrane is performed in two steps. First, the PC membrane is bonded to the top PDMS layer. The PDMS layer and PC membrane are washed with isopropyl alcohol (IPA) and deionized water (DI) and dried with compressed air. The 5% v/v aqueous APTES solution is prepared by mixing APTES (99%) reagent with EDI and heating at 80 °C. The porous membrane is activated by corona treatment, then, immersed in the APTES solution for 20 min. The PC membrane and the activated PDMS are brought into contact and, subsequently, pressed together. Second, the PDMS/PC bilayer is sealed to the bottom PDMS layer using corona activation and manual assembly. The entire structure is placed in a furnace at 90 °C to improve adhesion between layers. Before starting the experiment, the microsystems are autoclaved at 120 °C.

Animals: The animals were kept in aseptic conditions in an isolator and were regularly inspected to assess microbial and parasitological infections (including Helicobacter spp.). Three SCID mice were administered with 4 mg/L of dexamethasone sodium phosphate (Dex) (Merck, Lyon, France) via drinking water, as previously described [14], two weeks before euthanasia by carbon dioxide inhalation for tissue culture experiments. Experiments were conducted in the animal facility (PLETHA Pasteur) at the “Institut Pasteur de Lille” (research accreditation number A59107). The animal protocols were approved on 3 May 2011 by the French regional ethics committee (approval number CEEA 112011).

Colon explant preparation: The colon recovered from the animals was dissected and cleaned of fecal contents with cold Hank’s Balanced Salt Solution (Sigma, St. Louis, MI, USA) supplemented with penicillin (100 U/mL, Thermofisher, Waltham, MA, USA), streptomycin (100 µg/mL, Thermofisher, USA), and metronidazole (50 µg/mL, Sigma, USA). The tissue was opened along its length and cut into 8 mm^2^ pieces. The explants (9 mm^2^ in surface, 300 μm thick) were manually transferred into the device. Dulbecco’s modified Eagles medium (DMEM-F12, Sigma, USA) containing 10% fetal bovine serum (FBS), penicillin (100 U/mL), streptomycin (100 µg/mL), L-glutamine (2 mM), insulin/transferrin/selenite mix (1:100, Thermofisher, USA), Albumax (1 mg/mL, Life Technologies, Carlsbad, CA, USA), hydrocortisone (1 µM, Sigma, USA ‘), glucagon (14.3 nM, Sigma, USA), 3,3’,5’-triiodo-L-thyronine (1 nM, Sigma, USA), ascorbate-2-phosphate (200 µM, Sigma, USA), linoleic acid (20 µM, Sigma, USA), estradiol (10 nM, Sigma, USA), and keratinocyte growth factor (KGF) (50 ng/mL, Sigma, USA) were provided to the bottom microchannel of the four devices through the silicon tubing via the four-way MFCS equipment. The medium waste was collected in four independent tubes. The absence of bacterial contamination was screened by plating the medium onto culture media (Trypticase soy), for at least 72 h at 37 °C. Mouse colonic sections obtained immediately after dissection were used as controls for histology characterization.

Histological analysis: The cultured explants were stopped after 8 days of culture and, then, fixed in 10% formalin and embedded in paraffin. Sections of 5 µm were stained with hematoxylin/eosin and safranin (Leica Autostainer-XL, Rueil-Malmaison,, France). Sections stained with hematoxylin/eosin and safranin were examined using a Leica DMRB microscope equipped with a Leica digital camera connected to an Imaging Research MCID analysis system (MCID software, Cambridge, United Kingdom).
